# Using Virtual Reality to Reduce Anxiety and Improve Hospital Experience in Paediatric Orthopaedic Patients and Their Parents

**DOI:** 10.3390/children10081409

**Published:** 2023-08-18

**Authors:** Natasha Oh, Nina Parrish, In Woo Lee, Sasha Temple, Oliver Perkins, Michail Kokkinakis

**Affiliations:** 1 Faculty of Life Sciences and Medicine, King’s College London, London WC2R 2LS, UK; nina.parrish@kcl.ac.uk (N.P.); in_woo.lee@kcl.ac.uk (I.W.L.); michail.kokkinakis@gstt.nhs.uk (M.K.); 2 Paediatric Orthopaedic Department, Evelina London Children’s Hospital, London SE1 7EH, UK; sashanicole.temple@gmail.com (S.T.); oliver.perkins@gstt.nhs.uk (O.P.)

**Keywords:** anxiety, virtual reality, paediatric orthopaedics, hospital experience, pain

## Abstract

The hospital environment can be a stressful environment for paediatric patients and their parents, which is often characterised by heightened levels of pain and anxiety. To address these challenges, many innovative intervention methods has been explored. For example, immersive virtual reality (VR) headsets as a distraction method has become an increasingly popular intervention in recent years. This study aimed to evaluate the effectiveness of VR using ‘Rescape DR.VR Junior’ in reducing pain, anxiety, and enhancing the overall hospital experience for paediatric orthopaedic patients and their parents. A total of 64 patients aged 4–18 years were included in this study, which utilised a control group (interacting with a play specialist) and a VR intervention group (including pre-operative patients and fracture clinic patients). Anxiety and pain levels were measured using a 10-point Likert scale before and after the intervention, and validated questionnaires were used to assess parental anxiety and overall hospital experience. The results indicated that VR intervention significantly reduced patient and parental anxiety both before surgery and in the fracture clinic setting (*p* < 0.5). However, no significant reduction in pain scores was observed in either environments. Comparatively, VR intervention was found to be comparable to traditional play methods in terms of reducing anxiety in the pre-operative environment. All patients and parents agreed that the use of VR distraction methods significantly improved their hospital experience. In conclusion, VR is an effective method for reducing child and parental anxiety and enhancing the hospital experience and can be used alone or in conjunction with a play specialist.

## 1. Introduction

Hospitals can be an anxiety-inducing environment for many children, particularly those undergoing invasive procedures. More specifically, within the hospital setting, pre-operative anxiety on the ward before surgery is prevalent amongst many children and is typically maximal right before induction of general anaesthesia [1]. It can have both short- and long-term effects postoperatively. Anxious children pre-operatively are associated with higher incidences of increased intensity and level of pain and anxiety, poorer and prolonged recovery and negative behaviours post-operatively [2,3]. Anxious pre-operative children are often more agitated and less compliant during medical procedures which can increase post-operative morbidity and mortality [4] and resistance to treatment.

Having a child admitted to the hospital for surgery is a daunting experience for the parents, and both are at risk of post-traumatic stress symptoms (PTSS) [5,6]. Parental anxiety has also been proven to influence and increase their child’s pre-operative anxiety [3,7].

Waiting room anxiety, specifically the amount of time spent waiting can cause prior anxiety before consultation [8,9]. In our study, we particularly focused on orthopaedic patients and environments. One example is the fracture clinic—one of the most common situations a paediatric orthopaedic patient will encounter [10]. Anxiety is common in children before medical procedures, even when they are non-invasive. In the context of the fracture clinic, this includes plaster removal [11], plaster application, dressing changes and pre-and post-operative clinical and radiological assessments.

Distraction is a well-established and commonly used method during medical procedures [12], for example, the use of movies [13], music [14], books and games and even hospital clowns [15], all of which have been proven to reduce pain and anxiety [16]. Play specialists are commonly used today across many children’s hospitals and are an effective and well-established distraction method to help reduce hospital anxiety and pain [6].

The use of strategic play and various distraction tools to help a child cope with unpleasant medical procedures aids in minimising their fear and anxiety [17,18,19]. Play specialists also help prepare children and their families for procedures. They help them gain a better understanding of what the medical procedures involve and what treatment they will receive, using age- and developmental-appropriate techniques.

The use of VR as a distraction method has become an increasingly popular intervention in recent years [20]. VR distraction provides a truly immersive experience both visually and auditorily and is particularly engaging for children [21]. Paediatrics is an area that holds immense potential for the opportunity to use VR as it appeals directly to their imaginative and inquisitive nature. The children can orientate and interact with the 3D environment projected onto the headset screen right in front of their eyes. VR has been implemented in several paediatric and adult clinical settings and procedures which have been proven it as a successful distraction method. Examples include venepuncture [22], dental procedures [23], burn wound car [24], cancer treatment [25] and imaging procedures such as MRI scans [26].

Furthermore, for children with neurological conditions, the stressors associated with being in a hospital can be even more pronounced. Neurological conditions introduce an additional layer of complexity to the situation and children with such conditions often have unique medical needs, a heightened sensitivity to stimuli, and potential difficulties in communicating their feelings or understanding the changes around them [27]. These factors can intensify their stress and make the hospital environment particularly overwhelming. Therefore, the inherent enjoyment and engagement associated with the immersive nature of VR experiences can serve as powerful motivators for children with neurological disabilities, for example, in children with autism where previous research has demonstrated the benefits of VR [28,29]. This is compared to traditional therapeutic activities which might be met with resistance or disinterest.

The use of VR in paediatric orthopaedic patients and its effect on anxiety and pain in different orthopaedic environments has been only scarcely reported in the international literature [30,31,32,33].

The aim of this study is to compare the use of VR to standard hospital care which includes the use of play specialists. Currently, at Evelina Children’s Hospital paediatric orthopaedic department there is a team of health play specialists who support young children and adolescents along with their families through therapeutic play sessions. The orthopaedic ward has a play area, which provides a range of age-appropriate toys, activities, game consoles, books and DVD players.

The objectives of this study are as follows:

1. Compare the efficacy of virtual reality (VR) intervention to standard hospital care (involving play specialists) in reducing pain and anxiety levels before orthopaedic elective surgery;
2. Measure baseline pain and anxiety levels in the waiting room environment of the fracture clinic as well as evaluate the use of VR intervention in reducing pain and anxiety levels in the waiting room environment of the fracture clinic prior to the child’s consultation;
3. Evaluate the impact of VR intervention on parental anxiety;
4. Gather qualitative feedback to assess the overall effect of VR on the hospital experience for both children and their families, including practicality and recommendations for using the VR headset.

## 2. Materials and Methods

### 2.1. Inclusion and Exclusion Criteria

Children were eligible if they met the following inclusion criteria: (1) aged from 4 to 18 years old; and (2) visiting the fracture clinic for a follow-up (after undergoing a surgical procedure in the operating theatre or conservative treatment of their fracture with a plaster cast) or admitted to the hospital for an elective orthopaedic surgery.

The participants in the study consist of the typical and common group of patients presented in the paediatric fracture clinic or admitted in a regional tertiary referral centre for an elective paediatric orthopaedic procedure.

Exclusion criteria included the following: (1) a diagnosis of epilepsy or a history of seizures; (2) any sort of cognitive/neurological impairment preventing them from playing a VR game; and (3) patients with poor general health, i.e., suffering from cold, flu, headaches, etc., as these can increase susceptibility to adverse outcomes.

Eligible children along with their parents were informed about the study and verbal consent was obtained before data collection. The study was conducted in accordance with the Declaration of Helsinki and approved by the Institutional Review Board of Evelina Hospital Department of Clinical Governance (code number: 14620, date of approval: 20 January 2023).

Both the control group and intervention group had similar demographics and were controlled for age, sex and type of procedure. In both groups, there was adequate representation of males and females.

This was a prospective observational and comparative study conducted in two clinical settings.

Pre-operatively (comparative): VR intervention group vs. control group with Play Specialist Intervention (PSI).

- VR Intervention Group: This group comprised children who used and wore a virtual reality (VR) headset coupled with a control tablet (Samsung Galaxy Tab A 10.1).
- Control Group (Play Specialist Intervention—PSI): Children in this group received standard care facilitated by a play specialist, involving conventional distraction techniques such as games and toys.
- Timeline of Measurements: Anxiety and pain levels were measured in each group before and after the use of either the VR headset or the play specialist. All these measurements were carried out prior to the child’s surgical operation.

Fracture Clinic (prospective observational): VR intervention group with a baseline group.

The inclusion of the fracture clinic as an additional clinical domain for investigation aimed to explore the potential advantages of employing virtual reality (VR) intervention methods to alleviate waiting room anxiety before consultations. Waiting room anxiety is a prevalent concern in this context. Unlike the pre-operative group, where we established distinct control and intervention groups, the approach in the fracture clinic setting was more observational in nature. In the fracture clinic, the absence of a routine play specialist prompted us to compare anxiety and pain levels against baseline measures, which were obtained before the introduction of the VR intervention, which allowed us to see the potential benefits VR may have in paediatric waiting rooms. This observational methodology in the fracture clinic contrasts with the pre-operative group, where we rigorously implemented both control and intervention groups for a more controlled analysis.

- VR Intervention Group: This group comprised children who used and wore a virtual reality (VR) headset coupled with a control tablet.
- Baseline Control Group: Children in this group served as a baseline comparison and had no sort of routine intervention
- Timeline of Measurements: In the VR intervention group, pain and anxiety levels were measured before and after the VR intervention prior to their medical consultation. For acquiring baseline anxiety and pain levels, these measurements were conducted in the waiting room prior to their consultation.

Children in the pre-operative and fracture clinic settings were assigned to either the VR intervention group or the control group based on the day of data collection. The control PSI received standard care with the play specialist using commonly used distraction techniques (video game consoles, mobile tablet device, conventional toys, etc.).

### 2.2. The Virtual Reality Intervention

The DR.VR Junior Headset is a Class 1 Medical Device coupled with a Samsung Galaxy Tab A 10.1 control tablet created by Rescape Innovation LTD and provided to the Paediatric Orthopaedics department at Evelina’s Children Hospital. Rescape Innovation Ltd. operates as a biotechnology company with its headquarters located in Cardiff, Wales, United Kingdom. This innovation facilitates the swift, convenient, and trackable implementation of VR therapy in paediatric environments.

**Figure 1:**
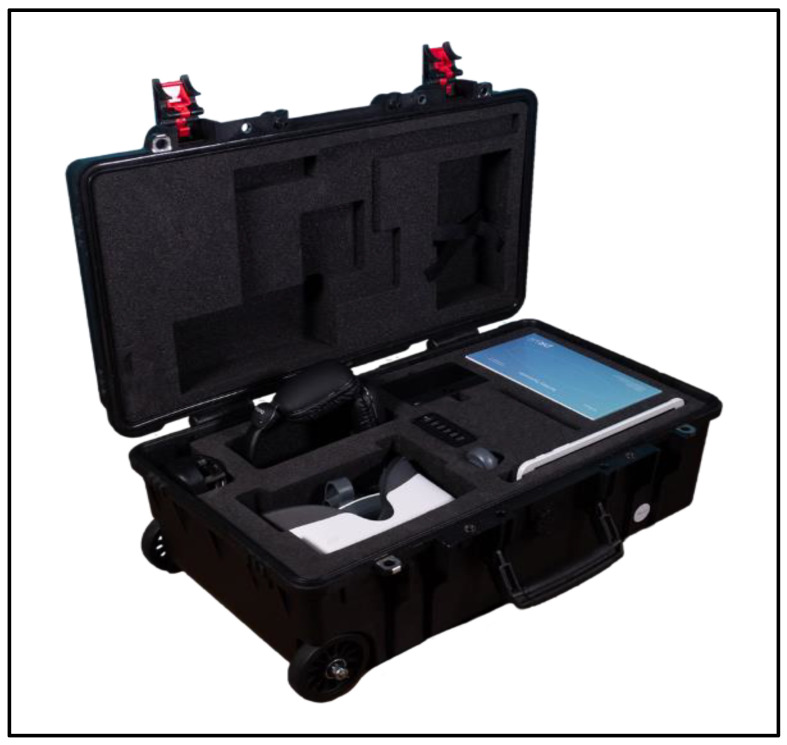
VR headset and case.

The DR.VR junior headset is designed to be used by any healthcare professional. Training on how to use the headset was provided by the company to both medical staff and medical students involved in this study. The headset is coupled with headphones, a control tablet that does not require external support including hospital Wi-Fi and a built-in charging unit within the case, as seen in Figure 1. It is completely independent and can be used anywhere.

**Figure 2:**
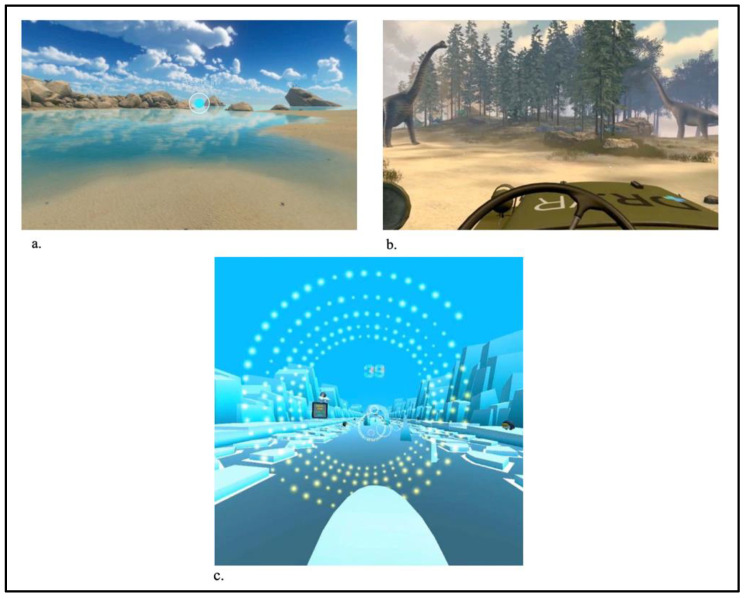
(**a**) Relax experience, (**b**) escape experience, and (**c**) distract experience.

The headset which is worn by the child has preloaded experiences built into it and includes several landscapes available for exploration alongside narration and guided tours such as underwater, space and dinosaur environments (Figure 2). Children also have the option to pick an immersive distraction game or three guided meditation and breathing exercises using a visual guide to help them breathe deeper and slower.

Putting the parent(s) at ease is an important healthcare objective and the DR.VR junior headset has been designed to do this. This Virtual Reality tool allows family members to be involved in their child’s VR experience by watching along on the control tablet provided. Each built-in programme on the headset has a duration of less than 15 min, which is the maximum formal industrial recommendation length of single use at a time.

### 2.3. Assessment Instruments

The primary outcome measures were pain and anxiety before and after intervention before orthopaedic elective surgery and in the fracture clinic. Secondary outcome measures included pre and post-VR intervention parental anxiety and qualitative feedback on the overall effect of VR on the family’s hospital experience including practicality and recommendation of the VR headset.

#### 2.3.1. Child Anxiety and Pain

**Figure 3:**
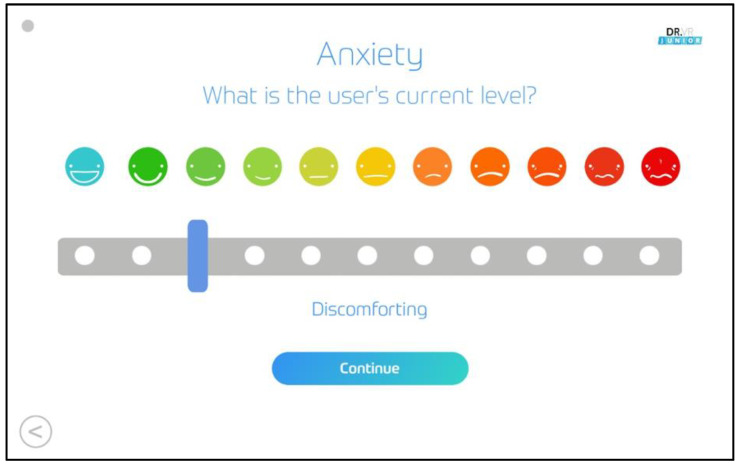
The 10-point Likert scale.

The primary outcome measure was child anxiety and pain which was measured using a 10-point Likert scale [34] (Figure 3).

#### 2.3.2. Parental Anxiety

**Figure 4:**
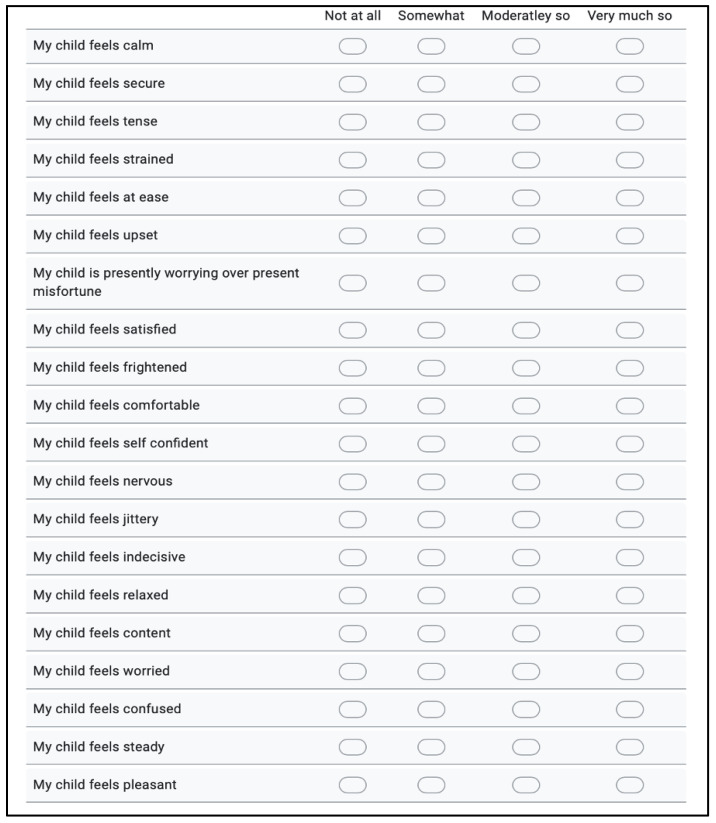
STAI-P for measuring parental anxiety.

Parental anxiety was measured using a State-Trait Anxiety Inventory parent version (STAI-P) (Figure 4) derived directly from the original STAI S-Anxiety Scale [35]. Parents completed the state form both before and after the use of the VR headset and PSI. This is a self-reporting instrument with 20 items each asking the user to rate on a 4-point Likert scale of 1 = not at all; 2 = somewhat; 3 = moderately so; and 4 = very much so. The 20 items consist of 10 anxiety-present questions, e.g., ‘My child feels frightened’ and 10 anxiety-absent questions, e.g., ‘My child feels calm’. Total scores range from 20 to 80, with higher scores indicating increased anxiety.

#### 2.3.3. Effect of VR on Hospital Experience

**Figure 5:**
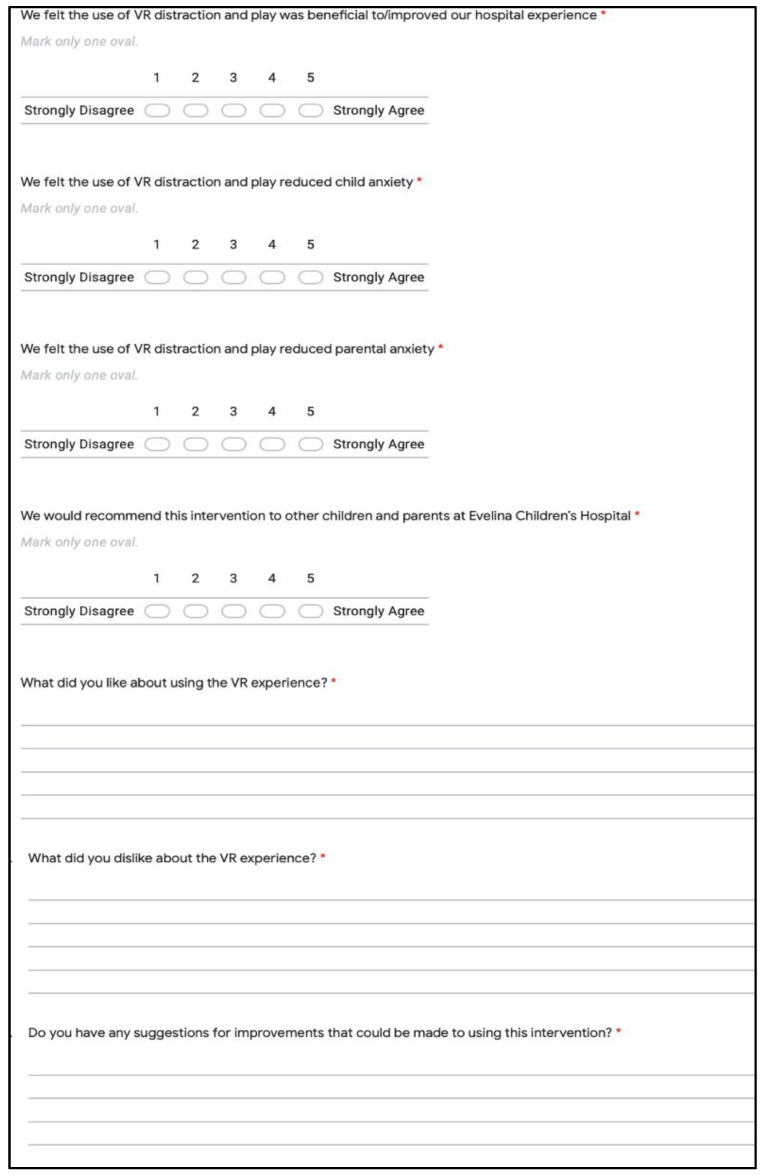
VR hospital experience form questions. (* indicates the question is mandatory).

Subjective feedback from the parents/guardian of the patients who used the VR headset using a questionnaire form on a tablet (Apple iPad) (Figure 5) stationed within the orthopaedics ward of Evelina Children’s Hospital was collected, which included 7 statements, 4 of which were scored on a 5-point Likert scale. Additionally, 3 open-ended questions were included to gather information on the patient’s preferences, dislikes, and suggestions for potential improvements in the VR experience.

### 2.4. Intervention Group

Demographic details were collected using the built-in system on the VR headset (age, gender). The built-in Likert scale on the headset device (Figure 2) was used to measure self-reported anxiety and pain (primary outcome measures) before intervention to establish a baseline (T0) and immediately after the VR intervention (T1). STAI-P was filled out at T0 and T1 by the parent. Furthermore, subjective feedback specific to the VR from both the child and parent were collected at T1 on a questionnaire for children in the intervention group. This was carried out both pre-operatively and in the fracture clinic. For the pre-operative patients, they were provided the VR intervention on the wards before they were transported to the operating theatre. In the fracture clinic, the VR intervention was provided before they were called into the consultation room.

### 2.5. Control Group

For the pre-operative patients, a separate control group questionnaire was designed which had copies of the Likert scale for pain and anxiety and the STAI-P for parental anxiety for both the VR as well as the PSI groups.

For children in the fracture clinic, baseline anxiety and pain were measured using the Likert scale alongside parental anxiety using the STAI-P in the waiting room before their consultation. These baseline scores were then used to compare against the children in the intervention group who used the Virtual Reality tool before their consultation.

### 2.6. Data Analysis

Statistical analysis was performed using GraphPad Prism software (Version 9.5.1). Normality testing for numerical data was carried out using the Shapiro–Wilk test. The normality testing results indicated that the data did not meet the assumptions for a parametric test. Therefore, a non-parametric test, specifically the Wilcoxon rank-sum test, also known as the Mann–Whitney U-test, was used to evaluate the significance of the differences between anxiety and pain scores before and after the VR/play specialist intervention and between the two different groups in the comparative study pre-operatively. Significance for all statistical tests was determined at *p* ≤ 0.05.

## 3. Results

A total of 64 children of both sexes were included, and all pre-operative children were aged between 13 and 18 and in the fracture clinic, they were aged between 4 and 15 in both the control and intervention groups. Table 1 shows a summary of the results and demographics for the 53 patients that were part of the intervention groups pre-operatively (VR and PSI) and the fracture clinic waiting room (VR).

### 3.1. Preoperative Group

One pre-operative patient chose not to use the VR headset and was not included in the study. In the pre-operative patient group, VR intervention was shown to significantly reduce anxiety by a mean of 2.48 points (*p* < 0.0001) with a standard deviation (SD) of 1.60. (Table 2). We observed a trend in pain reduction with VR, with pain scores reducing by a mean of 0.43 (*p* = 0.1250) and an SD of 0.51; however, this was not found to be statistically significant. It is important to note, however, that the majority of VR patients (16/23) reported 0 pain before the intervention and therefore a reduction would not be possible to observe.

We measured a significant reduction in parental anxiety following VR, with a mean reduction score of 12 (*p* = 0.0006) (Table 3).

Children who received PSI also demonstrated a statistically significant reduction in anxiety by a mean of 3.05 points on the Likert scale (*p* < 0.0001) with an SD of 2.16. As seen in the VR intervention group, a trend in pain reduction was observed, with pain reducing by a mean of 0.55 points (*p* = 0.1250) and an SD of 1.32; however, this was not statistically significant. Once again, as the majority of these patients (16/20) reported 0 pain before the play specialist intervention, a reduction in pain was not possible to observe.

For parental anxiety in the control play specialist group versus the VR intervention group, we found that both play specialist and VR interventions were comparable in their ability to decrease parental anxiety. The play specialist decreased parental anxiety by an average of 17.35 points, while the VR intervention decreased parental anxiety by an average of 17.44 points. There was no significant difference found between the extent to which each group reduced parental anxiety (*p* = 0.457).

### 3.2. Fracture Clinic Group

In the fracture clinic, we utilised an observational approach, in contrast to the pre-operative group. This distinction arises from the absence of routine play specialist intervention within the fracture clinic. Nonetheless, our methodology involved a meticulous collection and observation of baseline pain and anxiety levels in the waiting room. These baseline measurements served as a foundation against which we could compare the outcomes of the post-VR intervention in the intervention group. By adopting this analytical approach, we aimed to gain valuable insights into the potential effectiveness of VR in mitigating waiting room anxiety where we observed the following.

Two of the children’s parents chose not to use the VR due to the long waiting time, and so they were not included in the study.

In the fracture clinic patient group, VR significantly reduced anxiety by a mean of 1.00 point on the Likert scale (*p* = 0.0312) with an SD of 0.94. Mirroring our pre-operative results, we observed a trend in pain reduction post-intervention, with the scores reducing by a mean of 0.50 and an SD of 1.43; however, this was not statistically significant (*p* = 0.500). Unlike in the pre-operative context, there was not a majority of patients reporting no pain pre-intervention (only 5/10 reported 0 pain). We measured a noticeably significant reduction in parental anxiety following VR, with a mean reduction score of 23 (*p* = 0.0312).

### 3.3. Hospital Experience

All patients and their guardians who used the VR headset and filled out the hospital experience questionnaire in either environment ‘strongly agreed’ or ‘agreed’ that the use of VR distraction and play was beneficial to/improved their hospital experience, with nine patients (64%) choosing the option of strongly agree. Twelve patients (86%) said ‘strongly agree’ to recommending this intervention to other children and parents. The remaining two patients (14%) responded saying that they ‘agreed’ with this statement. All but one of the participants said they ‘strongly agreed’ or ‘agreed’ that VR reduced child anxiety, with nine patients (64%) saying they ‘strongly agreed’. The remaining one user reported that they ‘disagree’ with this statement. Finally, eight patients (57%) said they ‘strongly agree’ that the VR headset reduced parental anxiety, three patients (20%) chose ‘somewhat agree’ and one user reported they ‘disagree’ with the statement (Table 4).

### 3.4. Comparison between the Two Clinical Environments

The pre-operative patients reported a greater average anxiety level of 4.04 and 5.70 pre-intervention (VR or PSI, respectively), compared to an average of 1.8 and 2.64 in the pre-VR intervention and baseline control group, respectively, in the fracture clinic. Both environments demonstrated a statistically significant reduction in both child anxiety and parental anxiety post-VR, with a greater reduction in child anxiety seen pre-operatively. Parental anxiety was highest pre-operatively and showed the greatest reduction after play specialist intervention.

The fracture clinic reported a higher mean average of pain levels in the pre-intervention group and baseline in the control group, and a greater average reduction in pain post-VR intervention than pre-operatively. However, in both environments, the reduction in children’s pain was not found to be statistically significant. We believe that the results here hold clinical significance as, of those patients in both environments who reported a level of pain above 0 pre-intervention, 7/12 reported a reduction in pain post-VR intervention. The remainder reported no change. One patient who reported no pain pre-intervention did report an increase in pain by 2 on Likert scale; however, we hypothesise that this anomaly is due to the young age of this patient (4yr old- the youngest of all patients included in the study) and thus must have a possible misunderstanding of the Likert scale.

## 4. Discussion

### 4.1. Principal Findings

Extensive research has established that pre-operative anxiety, waiting room anxiety, and parental anxiety can have detrimental effects and exacerbate the perception of pain and anxiety within the hospital setting [4,8,36]. Despite the best efforts of healthcare professionals and the utilisation of conventional play techniques, helping children to forget their hospital surroundings remains a challenging task.

Our study has demonstrated that VR is an effective distraction method to reduce anxiety in paediatric orthopaedic patients and their parents/guardians across a range of ages in both a ward-based and waiting room environment. The majority of children exhibited some baseline anxiety, with older patients tending to experience lower baseline anxiety in the two environments which was expected. However, it was intriguing to observe that older children still actively chose to engage with the VR headset. This suggests the allure of VR novelty, and also highlights that it can benefit not only young children but individuals across a wide age spectrum as a viable distraction technique.

The study, however, showed no significant difference in pain pre- and post-VR in both environments. Initial levels reported were all on the lower end, especially in the fracture clinic, with only one patient reporting a level of 7 pre-operatively. VR may have a smaller impact on reducing pain that is minimal, especially in the fracture clinic, which is a low-risk environment. The relationship between pain and distraction appears to be grey; there are studies that have shown less pain after the VR intervention [23,37], whereas another study has shown no statistical difference in pain reduction after the intervention [38]. Thus, for further investigation, for example, choosing a clinical environment with increased baseline pain levels such as Accident & Emergency departments, will be interesting. Exploring the potential of VR in reducing pain in more challenging pain contexts could provide valuable insights into its effectiveness as an analgesic intervention.

Furthermore, the findings of this study suggest that combining VR with the presence of a play specialist could potentially yield even more significant reductions in both child and parental anxiety, and potentially pain as well. The synergistic effect of VR and the expertise of a play specialist may create a more immersive and tailored experience, thereby enhancing the overall therapeutic impact. However, it is worth noting that the standard routine utilisation of play specialists alone in our hospital setting has demonstrated effectiveness in reducing anxiety levels among both children and parents. This indicates that play specialists play a crucial role in alleviating anxiety and in promoting a positive hospital experience. Previous studies that have employed traditional distraction tools utilised by play specialist have indeed shown positive improvements in a child’s hospital experience [39,40]. While traditional play interventions led by play specialists have their merits and contribute to enhancing the hospital experience, the immersive and engaging nature of VR technology appears to offer a heightened level of distraction and emotional support.

Children’s engagement with play specialists can be influenced by a range of factors, including their temperament, age, prior experiences, and current emotional state. Some children might feel shy, anxious, or overwhelmed in the hospital environment, making it challenging for them to engage or sustain interactions. Additionally, the nature of their medical condition, pain levels, or emotional distress could impact their receptiveness to engagement [41].

In such instances, introducing alternative interventions like VR might offer a valuable solution. VR provides an immersive and novel experience that has the potential to capture the attention of even the most disengaged individuals. The interactive and visually stimulating nature of VR content could overcome initial reluctance, potentially leading to increased engagement and participation. In a systematic review and meta-analysis conducted just last year, the conclusive findings highlighted VR as a valuable and practical tool for significantly reducing self-reported pain and anxiety. Additionally, the study affirmed the utility of VR as an effective means of distraction during a range of medical procedures [41].

Consequently, while VR may offer additional benefits when used in conjunction with play specialists, it is important to acknowledge the ongoing value and efficacy of play specialist interventions as a standalone approach. Further research can explore the specific ways in which VR and play specialists can complement each other and optimise outcomes in clinical settings, ultimately improving the well-being of paediatric patients and their families.

The distraction option on the headset was the most popular choice (34 patients (64%) choosing the game) across all the ages, with 24 patients (71%) out of the 34 who chose the game showing a reduction in either pain/anxiety or both. For two of the younger patients (age 4), the game option was difficult for them to grasp despite picking the easier option and so, a second option was given, and both chose a guided landscape tour which was well received.

Positive subjective feedback from the users and other positive comments from healthcare professionals was received. All participants said there was nothing they disliked about the VR other than one who said it hurt her eyes after prolonged use. In terms of suggestions for improvement, most reported none, but some included “Would have liked it to be more available through the long stay in the hospital, which is boring at times” and “more viewing options”. Some also wished they could use it more often during their hospital stay.

### 4.2. Strengths

Strengths of this study included the use of a homogenous group and a universal standardised assessment tool (STAI-P), enhancing the reliability and comparability of anxiety measurements. Additionally, this study focused on a narrow range of surgical orthopaedic procedures, allowing for a more specific analysis of the intervention’s effectiveness.

Another strength was the absence of any reported delays or interruptions during the study, indicating seamless integration of the VR tool into both environments. This lack of interference with standard hospital care suggests that VR can be implemented as an additional distraction method alongside traditional techniques and play specialists, without hindering routine procedures.

### 4.3. Limitations

In the fracture clinic, it was observed that some parents were hesitant to use the VR headset, despite their child expressing interest, primarily because of the lengthy waiting time experienced in the clinic. This factor may have contributed to increased parental anxiety reported prior to the VR intervention. Additionally, language barriers posed challenges for certain parents, leading to their inability to complete the questionnaires.

We did not perform a power analysis to identify the sample number and certainly this can be considered in future study designs.

Another notable limitation pertains to the use of a singular self-report scale for evaluating both pain and anxiety in children. While this approach provides a practical and streamlined method for data collection, it does raise the possibility of overlapping or confounding responses. Using the same scale for assessing two distinct psychological constructs might inadvertently blur the nuances between pain and anxiety, potentially influencing the accuracy of our findings. Furthermore, relying on self-report scales introduces an inherent subjectivity to the data collection process. Children’s interpretations and expressions of their pain and anxiety levels might vary based on their understanding, personal perceptions, and communication abilities.

In light of these limitations, it is crucial to interpret the outcomes with a degree of caution. While the self-report scale offers valuable insights into the children’s experiences, supplementing it with complementary methods such as observer ratings or physiological measures in future research could enhance the comprehensiveness and robustness of our findings.

## 5. Conclusions

‘Rescape DR.VR’ headset is an effective distraction method, showing a statistically significant reduction in child and parental anxiety and improving the overall hospital experience in both orthopaedic environments in a wide age group. We also observed a trend in pain reduction following the VR intervention in the paediatric fracture clinic environment. The positive outcomes of this study therefore support the integration of VR technology as a beneficial adjunctive tool into paediatric orthopaedic settings.

## 6. Future Research

Larger sample sizes and a randomised control trial will enhance the validity of the results in future research. Based on our conclusions and results, we recommend further research to explore the effectiveness of VR in clinical areas such as paediatric A&E, anaesthetic room before induction, or during surgical procedures under local anaesthetic where children are kept awake. VR in the context of the radiology department, specifically prior to X-rays, CT scans and MRI scans, is also an area with few existing studies that has a large potential to reduce child anxiety. Furthermore, we need prospective studies to confirm not only the clinical effectiveness of the VR devices but also their cost effectiveness in different hospital environments to justify their purchase and maintenance, which is an important factor in the present context of increased health economic challenges. Cost effectiveness studies will not only justify the costs of buying the VR devices but also potentially facilitate significant savings by enhancing other clinical factors such as early discharge and pain relief. Whether the enhanced hospital experience with the use VR tools remains long term, with the children and their parents developing a more positive attitude towards hospital environments, is a very interesting hypothesis which can also be explored with future studies.

## Figures and Tables

**Table 1:** Summary of results for the intervention groups.

	Pre-Operatively		Fracture Clinic	Total
	VR Intervention	Play Specialist Intervention (Control)	VR Intervention	
Number of participants	23	20	10	53
Age range (years)	13–18		4–15	-
Mean age (years)	12.87	10.05	11.30	-
Anxiety score increased	0	0	0	0
Anxiety score decreased	15	16	6	37 (70%)
Anxiety score static/no anxiety	8	4	4	16 (30%)
Pain score increased	0	0	1	1 (2%)
Pain score decreased	4	4	3	11 (21%)
Pain score static/no anxiety	19	16	6	41 (77%)

**Table 2:** Mean child anxiety and pain.

	Fracture Clinic				Pre-Op VR Intervention			Pre-Op Control (Play Specialist Intervention)		
	Control	Pre VR	Post VR	Pre vs. Post	Pre VR	Post VR	Pre vs. Post	Pre	Post	Pre vs. Post
Anxiety	2.64	1.80	0.80	1.00	4.04	1.57	2.48	5.70	2.58	3.05
Pain	1.64	1.60	1.10	0.50	1.43	1.00	0.43	1.45	0.90	0.55

**Table 3:** Mean parental anxiety.

	Control (/80)	Pre-Intervention (/80)	Post-Intervention (/80)	DIFFERENCE Pre- vs. Post-VR
Fracture Clinic	35	44	21	23
Pre-Op (VR as intervention	N/A	41	29	12
Pre-Op (Play Specialist as intervention)	N/A	49	31	18

**Table 4:** Summary of responses for hospital experience.

	Strongly Disagree	Disagree	Somewhat Agree	Agree	Strongly Agree	Average Score
We felt the use of VR distraction and play was beneficial to/improved our hospital experience	0	0	0	5	9	4.64/5
We felt the use of VR distraction and play reduced child anxiety	0	1	0	4	9	4.50/5
We felt the use of VR distraction and play reduced parental anxiety	0	1	3	2	8	4.21/5
We would recommend this intervention to other children and parents at Evelina Children’s Hospital	0	0	0	2	12	4.86/5

## Data Availability

Data are unavailable due to privacy or ethical restrictions.
